# The Treatment of Inoperable Cancer

**Published:** 1902-08-09

**Authors:** 


### THE TREATMENT OF INOPERABLE CANCER.

In opening a discussion on the treatment of inoperable cancer, Mr. Henry Morris entered into a careful review of the various remedies and methods, other than the knife, which were employed with the object of curing, ameliorating, or retarding the disease, or of preventing relapse after its removal. He came to the following conclusions, among others: (*a*) That the serum treatment of malignant disease is not of the slightest use in carcinoma; that not one half of the cases of spindle-celled sarcoma disappear under treatment with Coley's fluid; that in cases of sarcoma other than spindle-celled Coley's fluid is not of value; and that the treatment by Coley's fluid has many dangers and should never be employed except in absolutely inoperable cases. (*b*) That Beatson's treatment is limited in its action to cases of mammary carcinoma and the local and glandular recurrences after mammary carcinoma, that only a small proportion of such cases are influenced by it, and that neither as a cure nor as a palliative can it be relied upon in any given case. (*c*) That in Finsen's light and in the X-rays, rodent ulcer has its most successful treatment, and that this is true not only of cases otherwise inoperable, but also of operable cases, in consequence of their excellent cosmetic results and of their effects upon non-evident foci of the disease. Nevertheless there are cases of rodent ulcer which resist these forms of treatment, and some of these cases may be successfully treated by excision and caustics. (*d*) That sarcoma, epithelioma and the other forms of carcinoma are best treated whenever possible by early excision; and that all forms of treatment hitherto tried in inoperable cancers of these kinds are uncertain and inconstant in their effects and untrustworthy as to the duration of the results which they produce, being in the vast majority of cases quite without palliative influence of any kind except possibly in relieving pain.

